# Chimerism in Wild Adult Populations of the Broadcast Spawning Coral *Acropora millepora* on the Great Barrier Reef

**DOI:** 10.1371/journal.pone.0007751

**Published:** 2009-11-04

**Authors:** Eneour Puill-Stephan, Bette L. Willis, Lynne van Herwerden, Madeleine J. H. van Oppen

**Affiliations:** 1 AIMS@JCU, James Cook University, Townsville, Queensland, Australia; 2 ARC Centre of Excellence for Coral Reef Studies and School of Marine and Tropical Biology, James Cook University, Townsville, Queensland, Australia; 3 Australian Institute of Marine Science, Townsville, Queensland, Australia; 4 Laboratoire Optimisation des Régulations Physiologiques, Université de Bretagne Occidentale, Brest, France; 5 Molecular Evolution and Ecology Laboratory, School of Marine and Tropical Biology, James Cook University, Townsville, Queensland, Australia; Northeastern University, United States of America

## Abstract

**Background:**

Chimeras are organisms containing tissues or cells of two or more genetically distinct individuals, and are known to exist in at least nine phyla of protists, plants, and animals. Although widespread and common in marine invertebrates, the extent of chimerism in wild populations of reef corals is unknown.

**Methodology/Principal Findings:**

The extent of chimerism was explored within two populations of a common coral, *Acropora millepora*, on the Great Barrier Reef, Australia, by using up to 12 polymorphic DNA microsatellite loci. At least 2% and 5% of Magnetic Island and Pelorus Island populations of *A. millepora*, respectively, were found to be chimeras (3% overall), based on conservative estimates. A slightly less conservative estimate indicated that 5% of colonies in each population were chimeras. These values are likely to be vast underestimates of the true extent of chimerism, as our sampling protocol was restricted to a maximum of eight branches per colony, while most colonies consist of hundreds of branches. Genotypes within chimeric corals showed high relatedness, indicating that genetic similarity is a prerequisite for long-term acceptance of non-self genotypes within coral colonies.

**Conclusions/Significance:**

While some brooding corals have been shown to form genetic chimeras in their early life history stages under experimental conditions, this study provides the first genetic evidence of the occurrence of coral chimeras in the wild and of chimerism in a broadcast spawning species. We hypothesize that chimerism is more widespread in corals than previously thought, and suggest that this has important implications for their resilience, potentially enhancing their capacity to compete for space and respond to stressors such as pathogen infection.

## Introduction

Chimeras are organisms containing tissues or cells of two or more genetically distinct individuals [1] which typically arise through fusion. Fusion of genetically distinct individuals has been documented in at least nine phyla of protists, plants and animals [2], [3], including cnidarians in experimental allorecognition studies [4]–[6]. However, the extent to which natural chimeras occur in populations of reef corals is currently unknown.

Natural chimerism provides both benefits and costs for genetically heterogeneous organisms [7]. A major benefit of chimerism is that colonies may have a greater store of genetic variability and hence a wider range of physiological qualities and characteristics compared to non-chimeric colonies [8]. Also, fusion provides a mechanism to increase in size more rapidly than growth alone, and could thus enhance chances of survival [9], [10]. Conversely, costs include potentially decreased growth rate and decreased reproductive output of fused colonies [11]. The occurrence of two (or more) genotypes within the same individual or colony could also lead to cell linage competition for position in the germ line [2], which has been identified as a potentially severe cost associated with the chimeric state [2]. The costs and benefits associated with chimerism have provoked considerable debate. While it is known that chimeras exist, their importance appears to be under-rated, primarily because chimerism challenges evolutionary theory developed for genetically homogeneous individuals and chimeras are commonly thought to be rare in natural populations [12].

Mutation within cell lineages (i.e., somatic mutation) is a second mechanism leading to the presence of genetically distinct tissues within individuals [2]. Somatic mutations are relatively common in plants, but usually only affect a portion of the meristem. Strictly speaking, such plants are chimeras as they are composed of two or more genetically distinct tissues and are indeed often referred to as “chimeras” [13]. To avoid confusion, however, we will refer to the latter as mosaicism rather than chimerism because of the various characteristics that clearly differentiate their respective origins, particularly the origin of chimeras through fusion versus the origin of mosaics through somatic mutation [14].

Natural chimeras usually originate from allogeneic fusions (i.e., fusions between different individuals of the same species). Although chimerism occurs in a wide range of organisms [2] and has even been recorded in humans and other mammals [15], [16], it has been reported much more frequently from the marine environment, primarily from benthic organisms with planktonic larvae or propagules, such as red algae [17] or colonial marine animals including corals, bryozoans and ascidians [18]. Thus, this phenomenon may be most common in species in which fragmentation and fusion are normal features of the life cycle [19]. Therefore, the occurrence of chimerism in colonial marine animals further challenges notions of genetic uniqueness within clonal organisms [20] and the commonly held view that clonality is a mechanism for maintaining well-adapted lineages [14].

Colonial, modular organisms, including most sponges, cnidarians, bryozoans, and many terrestrial and marine plants, are composed of repeated building units (modules such as polyps, zooids, etc.) that replicate through budding, and which lead to vegetative growth of colonies [21]. Within sessile, modular, marine invertebrates (e.g. sponges, bryozoans, ascidians, and cnidarians), chimeras can originate from the fusion of larvae that settle adjacently, or from the fusion of colonies that come into contact through growth or movement [22]. Because the allorecognition systems of adult colonial marine invertebrates generally effectively discriminate between clone mates and non-clone mates [23], low proportions of chimeras are typically expected in natural populations. However, studies have clearly demonstrated the possibility of genetically distinct corals fusing [4]–[6]. Also, the sometimes high occurrence of chimeras in natural populations of various colonial marine invertebrates other than corals [8], [22], [24]–[26] and under experimental conditions [27] indicates that their self-nonself recognition systems at least occasionally allow the fusion of genetically non-identical entities.

Chimeras have been widely observed in natural populations of colonial marine ascidians [8], [22], [24]–[26]; however, surprisingly little is known about the extent of chimerism in natural populations of adult corals. The majority of studies about chimerism in corals have focused on juveniles and particularly on larvae during the settlement phase when they come into contact with conspecifics. To date, aspects of chimerism have only been assessed in brooding corals [27]–[39], whereas coral reefs are dominated by broadcast spawning species of coral. Here we explore the extent of genetic chimera occurrence (i.e., the cohabitation of different genotypes within a single coral colony) within two populations of *Acropora millepora*, a common broadcast spawning coral on the Great Barrier Reef (Australia), using genetic characterization of coral tissues at 12 polymorphic DNA microsatellite loci.

## Results

### Proportion of chimerism in natural populations of Acropora millepora

A total of 984 samples, representing 124 colonies collected from populations of *A. millepora* at Magnetic Island and south-west Pelorus Island were genotyped using up to 12 microsatellite loci. All microsatellite loci used were highly polymorphic in both populations, and displayed up to 17 alleles (Table 1). Using conservative criteria (i.e., genotypes within colonies displayed two or more non-shared alleles), we estimate that 2% and 5% of *A. millepora* colonies in the Magnetic Island and Pelorus Island populations, respectively, are chimeras. In total, six chimeric colonies were observed. In the Magnetic Island population, 2 out of 59 colonies (colonies 56 and 59) displayed two genotypes that differed by one allele, and one colony (24) consisted of two genotypes differing by at least two alleles (Tables 2, 3). In the south-west Pelorus (Pelorus Island) population, three colonies (colonies 1, 44, and 15) displayed two genotypes that each differed by two or more alleles (Tables 2, 3). Overall, chimeras represent 3% of all sampled corals according to this conservative criterion (Table 2).

**Table 1:** Primer mix, associated microsatellites and dyes, concentrations, and number of alleles (*Na*) per population.

Primer mix name	Micorsatellite loci	Repeat motif	Associated WellRED dye	Concentration in 10x primer mix	*Na* in Magnetic Island population	*Na* in south-west Pelorus population
MP2	Amil2_006	(CA)_4_TA(CA)_4_	D2	0.8 µM	6	6
MP2	Amil5_028	(TCACA)_7_TCAC (TCACA)_4_ TCACTCACTCACA	D3	0.8 µM	8	7
MP2	Amil2_002	(TG)_10_	D4	0.28 µM	6	5
MP3	Apam3_166	(AAT)_28_	D2	1.5 µM	16	15
MP3	Amil2_22	(AC)_10_	D3	1.0 µM	13	14
MP3	Amil2_23	(AG)_7_	D4	0.6 µM	6	5
MP5	Amil2_010	TA(TG)_11_	D2	0.5 µM	17	14
MP5	Amil2_012	GA(CA)_6_GA(CA)_2_	D3	0.3 µM	3	3
MP9	Wgs_152*	(AT)_9_	D4	1.0 µM	12	8
MP9	Wgs_035*	(GTAT)_6_(GTTT)_8_	D3	1.5 µM	6	7
MP9	Wgs_189*	(ATCT)_7_	D2	2.0 µM	7	7
MP9	Wgs_134*	(GATA)_6_	D2	2.0 µM	5	4

*locus amplified only with chimeric samples.

**Table 2:** Chimeric colonies number and proportions (in %), and potential mosaics.

Population	Number of colonies with 1 allele difference, excluding potential somatic mutations	Number of colonies with ≥2 allele differences	Number of chimeric colonies	Proportion of chimeras within the population (if different at 1 allele, excluding potential somatic mutations)	Proportion of chimeras within the population (if different at ≥2 alleles)	Number of colonies with 1 allele difference potentially from somatic mutation = mosaic **
Magnetic Is (N = 59)	2	1	3	5%	2%	1 **
south-west Pelorus Is (N = 65)	0	3	3	5%	5%	0 **
All (N = 124)	2	4	6	5%	3%	1 **

Number and proportion (%) of chimeric colonies in two wild populations (Magnetic and south-west Pelorus Islands) of *Acropora millepora*, after excluding likely somatic mutations under the assumption of a stepwise mutation model. Potential mosaics (**) with a single allele difference potentially arising from a stepwise mutation are also shown (last column).

**Table 3:** Genotypes of the chimeric colonies.

Sample	Genotype proportion	Pop	Locus name	Amil2 012	Amil2 012	Amil2 010	Amil2 010	Amil2 002	Amil2 002	Amil5 028	Amil5 028	Amil2 006	Amil2 006	Amil2 023	Amil2 023	Amil2 022	Amil2 022	Apam3 166	Apam3 166	Wgs 152	Wgs 152	Wgs 134	Wgs 134	Wgs 189	Wgs 189	Wgs 035	Wgs 035
*Swp1a-d*	*50%*	*Swp*		*101*	*101*	*160* *	*172* *	*96*	*100* *	*132* *	*175*	*100*	*100*	*135*	*135*	*169*	*169*	*128* *	*131* *	*90*	*90*	*117*	*121* *	*168* *	*180* *	*170* *	*174* *
*Swp1e-h*	*50%*	*Swp*		*101*	*101*	*166* *	*168* *	*96*	*104* *	*175*	*175*	*100*	*102* **	*135*	*135*	*165* *	*169*	*158* *	*167* *	*90*	*102* *	*117*	*117*	*164* *	*196* *	*166* *	*194* *
**Swp15a-d,g,h**	**75%**	**Swp**		**101**	**103**	**160** *	**166**	**96**	**100**	**132**	**175**	**100**	**100**	**135**	**135**	**165**	**169**	**131**	**131**	**88**	**88**	**105**	**109**	**164**	**164**	**166**	**174**
*Swp15e*, *f*	*25%*	*Swp*		*101*	*103*	*164* *	*166*	*96*	*100*	*132*	*175*	*100*	*100*	*135*	*135*	*165*	*169*	*131*	*131*	*88*	*88*	*105*	*109*	*164*	*164*	*166*	*174*
**Swp44a-g**	**87.5%**	**Swp**		**101**	**101**	**166**	**166**	**96**	**96**	**175**	**180**	**100**	**100**	**135**	**137**	**171**	**183**	**155** *	**158**	**88**	**88**	**109**	**109**	**164**	**172**	**174**	**174**
*Swp44h*	*12.5%*	*Swp*		*101*	*101*	*166*	*166*	*96*	*96*	*175*	*180*	*100*	*100*	*135*	*137*	*171*	*183*	*158*	*161* *	*88*	*88*	*109*	*109*	*164*	*172*	*174*	*174*
**Swp64a,c-h**	**87.5%**	**Swp**		**101**	**101**	**160**	**166**	**96**	**100**	**132**	**132**	**96**	**100**	**135**	**135**	**163**	**167**	**158**	**158**	**88**	**96**	**117**	**117**	**180**	**180**	**174**	**174**
*Swp64b*	*12.5%*	*Swp*		*101*	*101*	*160*	*166*	*96*	*100*	*132*	*137* **	*96*	*100*	*135*	*135*	*163*	*167*	*158*	*158*	*88*	*96*	*117*	*117*	*180*	*180*	*174*	*174*
**Mag24a-g**	**87.5%**	**Mag**		**101**	**101**	**160**	**178** *	**96** *	**100**	**185** *	**185**	**98** *	**100**	**133**	**135**	**171** *	**171**	**155**	**158**	**92** *	**118** *	**109**	**117**	**164**	**176**	**178**	**178**
*Mag24h*	*12.5%*	*Mag*		*101*	*103* *	*160*	*166* *	*100*	*100*	*137* *	*137*	*100*	*102* *	*133*	*135*	*173* *	*179* *	*155*	*158*	*90* *	*104* *	*109*	*117*	*164*	*176*	*178*	*178*
*Mag56a*	*12.5%*	*Mag*		*101*	*101*	*160*	*166*	*96*	*96*	*137*	*175* *	*100*	*100*	*133*	*135*	*157*	*167*	*131*	*131*	*88*	*90*	*109*	*109*	*164*	*164*	*182*	*182*
**Mag56b-h**	**87.5%**	**Mag**		**101**	**101**	**160**	**166**	**96**	**96**	**137**	**137**	**100**	**100**	**133**	**135**	**157**	**167**	**131**	**131**	**88**	**90**	**109**	**109**	**164**	**164**	**182**	**182**
**Mag59b-h**	**87.5%**	**Mag**		**101**	**101**	**158**	**166**	**96**	**100**	**175**	**175**	**100**	**100**	**135**	**135**	**163**	**177**	**131**	**131**	**88**	**92**	**117**	**121**	**164**	**180**	**170**	**174**
*Mag59a*	*12.5%*	*Mag*		*101*	*101*	*158*	*166*	*96*	*100*	*132* *	*175*	*100*	*100*	*135*	*135*	*163*	*177*	*131*	*131*	*88*	*92*	*117*	*121*	*164*	*180*	*170*	*174*

Twelve loci genotypes of chimeric colonies for two wild populations (Pop): south-west Pelorus (Swp) and Magnetic Island (Mag). The most common genotype per colony is indicated in **bold**. The proportion (in %) of each genotype within the colony is estimated based on the number of branches with the same genotype. Samples are named according to: their site of collection (Mag or Swp), their colony number (1 to 59 in Magnetic Island, and 1 to 69 in south-west Pelorus Island), and the branch replicate (from A to H).

*non shared alleles (i.e., allelic differences).

**single allelic variation potentially arising from somatic mutations (N.B. Colony Swp 64 is not considered to be a chimera because the only allelic variation potentially arises from somatic mutation).

A less conservative estimate, based on counts of all colonies with more than one distinct genotype within a colony, including those that displayed just one non-shared allele (excluding single allele difference by one mutational step), indicates that 5% of colonies in both populations were chimeras. Mosaics arise from somatic mutations while chimeras originate from the fusion of genetically different individuals. Because the most common somatic mutation is a single mutational step, a colony displaying a single non-shared allele differing by only one mutational step, is more likely to be a mosaic (e.g. Swp 64, Table 2, 3). Based on this criterion, only 0.8% of the sampled colonies were potential mosaics (i.e., 1 colony out of 124). However, non-single step mutations may also arise through somatic mutations, thus colonies with a single non-shared allele could also represent cases of somatic mutations (e.g., Mag 56 & 59). If all single allelic differences were assumed to arise through somatic mutations, 2.4% of the sampled colonies potentially represent mosaics. Overall, however, we consider it highly unlikely that two somatic mutations could arise in relatively young coral colonies (<40 cm in diameter), and thus our conservative estimate of chimerism based on two non-shared alleles should avoid scoring colonies as chimeras that were in fact mosaics.

### Relatedness

Based on genotypes from eight loci, genetic relatedness among samples within chimeric colonies was high (mean QG = 0.654±0.160, Table 4). In contrast to the high relatedness found for genetically distinct branches within chimeric colonies, the vast majority of colonies within each population were unrelated (mean QG = −0.007±0.002). Two exceptions were neighboring clone mates in the Magnetic Island population (i.e. colonies Mag9 and Mag10, Mag16 and Mag17). Overall, fewer than 0.2% of paired samples (n = 8515 pairs) showed relatedness indices greater than average relatedness indices found for branches within chimeras (QG≥0.654±0.160, Table 4). Moreover, relatedness between rejecting colonies (Swp68 & Swp69) was very low, QG = 0.08. Hence, visually incompatible genotypes displayed low relatedness and clear genetic differences. Note that only 2 rejecting colonies were sampled (i.e., only one QG calculated) and more sampling would be needed to confirm the low QG value calculated when colonies are incompatible.

**Table 4:** Pairwise relatedness in chimeric colonies (bolded), in rejecting colonies (italicized), and in all samples.

Paired samples	Queller and Goodnight (1989) estimator - Mean
**Swp1a-d/Swp1e-h**	**0.289**
**Swp15a-d, g-h/Swp15e-f**	**0.884**
**Swp44a-g/Swp44h**	**0.898**
**Mag24a-g/Mag24h**	**0.026**
**Mag56a/Mag56b-h**	**0.920**
**Mag59a/Mag59b-h**	**0.907**
**All chimeras (n = 6 pairwise comparisons)**	**0.654±0.160**
*Swp68a-d/Swp69a-d*	*0.080*
All samples (n = 8515 pairwise comparisons)	−0.007±0.002

Comparisons of pairwise relatedness in chimeric colonies (**bolded**), in rejecting colonies (*italicized*), and in all samples. Pairwise relatedness estimators calculated according to Queller and Goodnight (1989).

## Discussion

High levels of chimerism (5% overall, or 3% according to a more conservative estimate based on the presence of at least two non-shared alleles) were found in two wild populations of the broadcast spawning coral, *Acropora millepora*, on the Great Barrier Reef. Both the Magnetic Island and the south-west Pelorus Island populations had similar levels of chimerism, i.e., 5% chimerism within each population based on genotypic differences at one allele, and 2% or 5%, respectively, based on genotypes displaying at least two non-shared alleles. These results indicate that chimerism is a common feature of this coral's biology.

Coral colonies that contain different genotypes may also arise through somatic mutation and therefore, based on this mode of origin, are best described as mosaics. Using the presence of a single non-shared allele differing by only one mutational step as the criterion for identifying mosaics, 0.8% of the sampled colonies were potentially mosaics while 3% were likely to be chimeras (with genotypes displaying at least two non-shared alleles). Thus chimeras represented a much greater proportion of colonies found to be genetically variable within the two study populations than mosaics.

Genetic chimerism has not been described for any wild population of coral prior to this study, but brooding corals under experimental conditions are known to have the potential to form genetic chimeras in their early life stages [27]. The application of molecular tools to studies of non-cnidarian colonial marine invertebrates has also revealed relatively high levels of chimerism within wild populations. Random Amplified Polymorphism DNA (RAPD) analysis assessed the presence and the extent of chimerism in the colonial ascidian, *Diplosoma listerianum* [24], and revealed that 34% of *Diplosoma listerianum* colonies in a wild population on the Langness Peninsula, Isle of Man (British Isles) possessed multiple genotypes (i.e., were chimeras). A similar study of one population from artificial settlement plates and seven natural populations of *Diplosoma listerianum* in the Isle of Man, North Wales, Cornwall and Devon (UK) also revealed high levels of chimerism [22]. In this latter study, chimeric colonies were present in all populations studied, at frequencies ranging from 3% to 61%, and up to six different genotypes were present in some colonies. The use of highly polymorphic microsatellite loci in two different populations of the ascidian, *Botryllus schlosseri* (one native population from Caesarea (Israel) and one recently introduced population from Woods Hole marina (MA, USA)) revealed ∼9% of colonies were chimeric in these two widely separated populations [26]. Molecular tools have been integral to investigate the presence of chimeras in natural populations of colonial marine invertebrates and sometimes reveal very high levels of chimerism. High levels of chimerism (up to 61%) in *D. listerianum* were probably uncovered due to the high intensity of sampling: 288 colonies, and 12 samples per colony, for relatively small sized colonies [22].

The proportions of chimerism in populations of *A. millepora* presented here are likely to underestimate the true extent of chimerism, as the sampling protocol was restricted to a maximum of eight branches per colony (Great Barrier Marine Park Authority permit limitations). Despite the small sample size per colony, we nevertheless documented up to two genotypes per colony (see Table 3). Given that an adult colony of *A. millepora* 40 cm in diameter consists of approximately 600 branches (pers. obs.), much larger sample sizes would have significantly increased our ability to detect chimerism. Moreover, if chimeric genotypes are cryptic within colonies, as our data suggest (see below), it is highly likely that our sampling missed a significant proportion of chimeras. Additionally, if chimerism reduces colony survival in the early stages of a coral's life, sampling relatively large colonies (from 15 to 40 cm in diameter) might have further underestimated the incidence of chimerism in the sample populations. On the other hand, if chimerism enhances colony survival, sampling relatively large colonies (from 15 to 40 cm in diameter) might have overestimated chimerism. Unfortunately, no data on the survival of chimeras are available to assess if the size class sampled is likely to have over- or under-estimated the occurrence of chimerism in the two populations. However, biases in our sampling protocol - i.e. avoiding multi-colour or non-uniform colonies, sampling only 8 branches per colony, and restricting sampling to relatively large colonies, and relatively small geographic scales within each population – is most likely to have under-estimated the extent of chimerism in the two populations of *A. millepora*.

Chimeric colonies of *A. millepora* had one dominant genotype and a second, cryptic genotype (Table 3). In the majority of the colonies (except colony Swp1), six or seven of the eight sampled branches were genetically identical and one or two were different. Such differences may reflect cell lineage competition where one genotype is morphologically resorbed, as described for *B. schlosseri* [40]. However, even after complete morphological resorption, the germ line and/or the somatic lineages of the inferior partner may still successfully parasitize the “winning” partner [40], [41]. Although morphological resorption is a possible explanation for “dominant” genotypes within chimeras, it has only been observed in cytomictical chimeras, which are defined as chimeras in which some cells of the two parent organisms have become so mixed that they can no longer be separated into individuals [1]. An example is the colonial tunicate *Botryllus schlosseri*, where fusion establishes a common circulation system which mixes blood cells from each partner of the chimera. In contrast, suspected coral chimeras in the wild (e.g. *Stylophora pistillata*) show no evidence of mixed cellular elements, as evidenced for instance by each partner retaining its original colour [1]. Coral chimeras are therefore more commonly referred to as “sectorial chimeras”, where each partner within the chimera remains an individual [1].

While this and other studies [26] may have underestimated the proportion of chimeras in wild invertebrate populations, strong theoretical arguments exist to support the hypothesis that chimeras are rare. Strassmann and Queller [14] highlighted the destructive genetic conflicts that can arise within chimeras. In particular, costs such as cell lineage competition are associated with the formation of chimeras [11], [28], [42]–[45]. However, other authors point out potential benefits associated with the chimeric state. Because chimeras harbour a greater genetic diversity than genetically homogeneous individuals, they can display “chimeric vigour”, i.e., they may be able to use or cope with a wider range of environmental conditions. Other benefits of chimerism include developmental synergism (i.e., two aberrant forms are able to produce normal structures in a chimera), optimization of mate location, and the advantage of larger size in size-specific ecological processes [2]. Specifically, fusion provides a mechanism for increasing size more rapidly than growth alone [46], and could thus increase chances of survival [9], [10] for species where survivorship is size dependent [1]. The benefit of harbouring higher genetic diversity and variability, and thus the ability to cope with more diverse environmental conditions, has been shown for the ascidian, *B. schlosseri* [41]. In this species, the somatic constituents of a chimera can be shifted from one genotype to another in response to environmental conditions (e.g., sea water temperature), indicating that some chimeras have the ability to “fine-tune” their genotype at critical times [41]. Controversies over the potential costs and/or benefits of the chimeric state primarily reflect difficulties in studying chimeras in different organisms, as many studies have been laboratory based and laboratory “forced” chimeras could lead to associations between incompatible individuals [2]. Future research should focus on investigating these questions in natural populations of chimeras.

Chimeras can originate from the fusion of larvae that settle close to each other or from the fusion of colonies that come into contact while growing or after movement [22]. Recent studies have shown that juvenile cnidarians are able to form chimeras under experimental conditions [27], and that fusion between allogeneic juveniles is promoted by the gregarious settlement of larvae [27], [38], which occurs commonly for a number of coral species [10], [28], [47]–[49]. Furthermore, if larvae of colonial marine invertebrates tend to aggregate with closely related individuals, they should be more prone to accept each other and fuse. The ascidian, *Botryllus schlosseri*, showed strong aggregation with sibling colonies, while unrelated colonies were significantly over-dispersed [50]. Larvae which shared a histocompatibility allele settled in aggregations and then promoted the formation of stable chimeric colonies in the field. Consequently, kin aggregations on limited available substrate could be one of the main causes of chimera formation in corals and other colonial marine invertebrates. Kin aggregations might be of even greater importance in broadcast spawning corals where thousands to millions of related juveniles are produced due to the often high synchrony in gamete release of adjacent colonies [51]. These related larvae may remain aggregated in the dense spawning slicks that form during still weather conditions [52] and subsequently reach settlement competency at the same time. We found high relatedness between genotypes within chimeric colonies, while relatedness among neighbouring colonies within populations or between rejecting colonies (e.g. Swp68 and Swp69) was close to zero. The high relatedness between genotypes within chimeric colonies suggests that coral planulae settle in kin aggregations and may subsequently fuse and form chimeras. Alternatively, it is possible that non-related larvae settle and fuse to form chimeras, but that only closely related individuals survive and maintain a chimeric state.

Another possible cause of chimera formation is the “window in ontogeny” as proposed by Rinkevich [53]. Natural chimerism originates during pregnancy in humans (blood chimeras, whole body, foetal-maternal, germ cell, and tumor chimeras) [16]. Similarly, a narrow window early in the ontogeny of colonial marine invertebrates, prior to the development of the allorecognition system, may allow the formation of chimeric entities [53]. Many marine invertebrates require days to months to reach a mature state of allorecognition. For example, maturation of the allorecognition system occurs within the first two weeks after metamorphosis in the hydrozoan *Hydractinia symbiolongicarpus* [54], but requires more than two weeks in the bryozoan *Celleporella hyalina* [55], and approximately four months post-settlement for the corals *Stylophora pistillata* [30] and *Seriatopora* spp. [37]. The lack of an efficient allorecognition system in the early stages of ontogeny in scleractinian and soft corals is believed to be universal, and juvenile chimeras may represent a case of allorecognition “failure”, promoted by the gregarious settlement of larvae that is characteristic of many cnidarians [38].

In summary, chimerism in corals may originate in their early life history stages. Indeed, kin aggregations of larvae have the potential to fuse, more so during the period when corals appear to lack an efficient allorecognition system. Following an initial chimeric state (bi- or multi-partner chimeras), maturation of the allorecognition system of corals could potentially lead to the death of the entire entity or of just some of the genotypes within the genetically heterogeneous individual. Alternatively, some genotypes could be rejected, or cohabitation of closely related individuals in a chimeric state could persist. In this study, we found high levels (3–5% overall) of chimerism in two wild populations of the spawning coral, *A. millepora*, in the central Great Barrier Reef. We also found that partners within a chimera were closely related in comparison to a lack of relatedness generally found among neighboring colonies.

These results constitute the first genetic proof of the occurrence of chimeras within wild populations of adult corals. One implication of these results is that multiple samples should be collected from coral colonies in studies characterizing the genetic structure of coral populations. In order to further elucidate current understanding about how chimerism arises and why it persists, future research should compare the fate of genetically homogeneous and chimeric corals exposed to various external stressors, such as increased water temperature, low salinity, or pathogens and microbes.

## Materials and Methods

### Sampling

To estimate the frequency of occurrence of chimeras in natural populations of the branching coral *Acropora millepora*, 65 colonies were tagged, photographed and sampled at south-west Pelorus Island (Pelorus Island, Palm Island group, S 18°33.030′ E 146°29.316′), and 59 colonies in Nelly Bay at Magnetic Island (Magnetic Island, S 19°10.115′ E 146°51.006′). Colonies between 15 and 40 cm in diameter were selected haphazardly for tagging from within an area of ∼10 m×400 m in Nelly Bay (Magnetic Island) and ∼10 m×200 m in south-west Pelorus (Pelorus Island). Fifteen cm was selected as the minimum size because colonies needed to be sexually mature for reproduction experiments and 40 cm was the maximum size sampled due to permit restrictions (Great Barrier Reef Marine Park Authority permit #G07/22554.1). Colonies that showed visual evidence of genetic differences, such as rejection lines or different morphological types or colors within an apparently single colony were excluded from sampling because it could not be discounted that such colonies represented two separate colonies in close association. Such colonies would have been scored as chimeras, whereas they represented cases of 2 (or more) incompatible colonies in close contact (e.g., swp#68 and 69, see below). The application of these conservative criteria means that it is likely that we missed some chimeras. Hence, this study provides a minimum estimate of the frequency of chimeras in natural populations of *A. millepora*. Sampling of one apparently fused colony, which appeared to be a single colony but had two clearly distinct colored sections separated by a rejection line, provided an opportunity to estimate what level of genetic difference resulted in rejection between two closely associated colonies. We considered this colony as two different colonies: Swp68 and Swp69.

In order to increase the likelihood of detecting genetic variability at the colony level, branches were sampled as far away from each other as possible across the colony. Because of permit restrictions, the maximum sample size per colony could not exceed eight branches. Samples were named according to (1) their site of origin and called Mag or Swp for Magnetic Island or south-west Pelorus Island respectively, (2) their colony number (1 to 59 in Magnetic Island, and 1 to 69 in south-west Pelorus Island), and (3) the branch replicate (from A to H). Once sampled, coral fragments were preserved and stored in 100% ethanol for down-stream DNA extraction and genotyping.

### DNA extraction & Genotyping

DNA was extracted using ‘Wayne's method’ [56]. DNA pellets were re-suspended in 200 mL of 10 mM Tris (pH = 9) and stored at 4°C. Prior to amplification, DNA was diluted at 1∶10 in MilliQ-water. Microsatellite loci were amplified in 10 µL multiplex PCR reactions, in PTC-100 Peltier Thermal Cyclers. Four different primer mixes (MP2, MP3, MP5, and MP9 see Table 1) each amplifying two, three or four microsatellite loci were used. Eleven microsatellites were specifically designed for *A. millepora* [57], [58]. Another locus (Apam3_166) previously developed for an acroporid species from the Caribbean, *Acropora palmata*, was also used because of its successful amplification in *A. millepora* and its high level of polymorphism [57]. These loci are unlinked [57], [58]. Reactions contained 1 µL DNA template, 1 µL 10x primer mix, 5 µL 2x Qiagen multiplex PCR mix, and 3 µL milliQ-water. The cycling protocol was: 1× 95°C (15 min), 35× (30 sec at 94°C, 90 sec at 50°C, and 60 sec at 72°C), 1× 60°C (30 min), and 4°C for ever. PCR products were diluted in Sample Loading Solution (SLS from Beckman Coulter) at 1∶10. Then, 2.5 µL of the diluted PCR products were loaded into a Genetic Analysis System CEQ 8800, together with 37.25 µL of SLS and 0.25 µL of 400 bp size standard (Beckman Coulter), for separation and subsequent PCR product size determination.

### Scoring

Once samples were run through the CEQ 8800, data were analyzed with the Fragment Analysis software from the Genetic Analysis System CEQ 8800 from Beckman Coulter (400FragmentAnalysisParameter). All results were scored manually. Based on No Template Controls peak values, peaks under 5000 RFU were not scored. Fragment sizes were recorded into Microsoft Excel for further analysis.

Eight loci (from primer mixes MP2, MP3 and MP5, see Table 1) were amplified and scored for all samples. In order to minimize scoring errors, all chimeric samples were processed twice. Four loci from primer mix MP9 (see Table 1) were only amplified and scored for chimeric samples.

### Chimerism

Several mutational models have been developed for microsatellites [59]: the Infinite Allele Model, Stepwise Mutation Model, Two phase model and Generalized stepwise model, and the K-allele model [60]. However, stepwise mutations, consisting of the addition or subtraction of one single repeat unit, are the most common mutations in microsatellite loci in plants, birds and humans [61]. Thus, in our study, when genotypes within a single colony differed by one allele at only one locus, we assumed alleles with a single repeat difference were probably produced by a somatic mutation, and therefore the colony could not be classified as a chimera (e.g., Swp64, Table 3). This approach provided a lower estimate of chimera proportions within the studied samples. Estimates of the rate of somatic mutations per locus per cell generation (10^−7^) for multicellular clonal organisms (e.g., *Goniastrea aspera*, *G. favulus*, and *Platygyrus sinensis*) [62] suggest it is far less likely that two independent somatic mutations would have occurred in the same tissue. We are therefore confident that if genotypes within a single colony displayed at least two non-shared alleles, the colony was chimeric. Consequently, a second, more conservative estimate of chimerism was calculated, where a colony had to display at least two non-shared alleles to be classified as a chimera. The percentage of chimerism was calculated within each population and overall, by determining the number of chimeric colonies compared to the total number of sampled colonies.

### Analysis

Microsatellite locus polymorphisms were calculated with GenAlEx 6.1 [63] within each population. Relatedness between all genotypes (based on eight loci) was calculated with the Queller and Goodnight estimator in GenAlEx 6.1 [63]. Queller and Goodnight's pairwise relatedness estimator (QG) values are expected to be equal or higher than 0.5 (i.e. QG≥0.5) for full sibs. Half sibs are expected to have values around 0.25, and QGs of unrelated individuals are expected to be close to 0 [64]. Relatedness analysis was performed on genotypes based on eight loci (see Scoring section) because these loci were amplified for all samples, while four extra loci were amplified only for chimeric samples, and resulting relatedness data were not comparable to the population level.
